# Mr. Humpage's Case of Tumefied Labium

**Published:** 1801-01

**Authors:** Edward Humpage

**Affiliations:** Surg. &c. Stroud, Glostershire


### *To the Editors of the Medical and Phyſical Journal.*

Gentlemen,

Being engaged in a large ſhare of Midwifery, and having delivered near two thouſand women, the caſe I am to relate never occurred to me till very lately: As I do not recollect to have ſeen or heard of ſuch an one, the inſertion of it in your valuable publication may not prove unacceptable. It may be a means of ſaving the young accoucheur a good deal of embarraſsment to be informed, that ſuch an occurrence will ſeldom cauſe any impediment to delivery, and that in no very great length of time the iſſue of it will be favourable. At a village near this place, I was called to deliver a woman of her ſeventh child, and who, I found, had been in labour ſome hours. Upon taking the neceſſary ſteps of information, I diſcovered the right labium different from what it ſhould be, and enough to excite my attention; but not conſidering this had any thing to do with the preſent buſineſs, I went on with the examination per vaginam. The os uteri was ſufficiently dilated to admit a part of the child's head into the pelvis. As the labour advanced, I had the opportunity of informing myſelf of the ſituation of the tumefied labium, which to my aſtoniſhment continued to increaſe. At length it acquired the ſize of a child's head juſt born, and became very tenſe, hard, and painful. About this period a conſiderable hæmorrhage took place, amounting in quantity, as far as I could judge, to a quart or more. I now began to be alarmed; and anxious to ſatisfy myſelf of this uncommon appearance of things, I made a more minute inveſtigation, and ſaw the true ſtate of the tumour; found it had burſted, aſſumed a dark livid hue, and that the bulk was not ſo much diminiſhed as we ought to have have expected from the quantity of blood thrown out, and which evidently came from that quarter. The labour not advancing in proportion to the smartness of the pains, I suspected the pressure of the distended labia might prove the obstacle.—Upon which I was led, as well as from other circumstances, to support the tumour, and confine it in the best manner I was able to one side, so as to give as much room as the nature of it would admit, for the passage of the child's head. The labour was accomplished, and with much greater ease than I had reason to suppose. It may be right to notice a circumstance respecting both mother and child, that the one had a well formed pelvis, and that the head of the other was of a moderate size; the woman likewise was robust and in full health. The length of time I was taken up with this patient, did not exceed much more than an hour, so that the accumulation of blood in the cellular membrane could not have been long collecting. As soon the woman was settled in bed, she expressed more uneasiness from the weight of the tumour than from any thing else. The common applications that such accidents indicate, were applied, with a full direction to support the part in as convenient a way as could be thought of. The discharge continued for a few days in large quantities, and very offensive. On the twelfth day the labia had nearly got to its natural size; the discharge became more purulent, and at length the cure was compleated without any difficulty whatever. The constitution never suffered in the smallest degree from this local complaint. Why a rupture of a vessel should happen here, and why it does not oftener occur, is not my design to inquire. I am more inclined to suggest a remedy, which I confess I should be disposed to adopt, was a similar case ever to fall to my lot again; I mean, the scarifying the parts with freedom immediately on the coming on of the accident, with the view of preventing the great distention of the teguments and its consequences; but how far this would be conducive to the end designed, experience alone can decide. I am,

GENTLEMEN,

Your humble servant,

EDWARD HUMPAGE, Surg. &c.

